# The complexity of the calretinin-expressing progenitors in the human cerebral cortex

**DOI:** 10.3389/fnana.2014.00082

**Published:** 2014-08-13

**Authors:** Nevena V. Radonjić, Juan A. Ortega, Fani Memi, Krista Dionne, Igor Jakovcevski, Nada Zecevic

**Affiliations:** ^1^Department of Neuroscience, University of Connecticut Health CenterFarmington, CT, USA; ^2^Institute of Medical and Clinical Biochemistry, School of Medicine, University of BelgradeBelgrade, Serbia; ^3^Experimental Neurophysiology, University Hospital CologneKöln, Germany; ^4^Experimental Neurophysiology, German Center for Neurodegenerative DiseasesBonn, Germany

**Keywords:** cortical interneurons, cortical development, transcription factors, Gsx2, GABA

## Abstract

The complex structure and function of the cerebral cortex critically depend on the balance of excitation and inhibition provided by the pyramidal projection neurons and GABAergic interneurons, respectively. The calretinin-expressing (CalR^+^) cell is a subtype of GABAergic cortical interneurons that is more prevalent in humans than in rodents. In rodents, CalR^+^ interneurons originate in the caudal ganglionic eminence (CGE) from Gsx2^+^ progenitors, but in humans it has been suggested that a subpopulation of CalR^+^ cells can also be generated in the cortical ventricular/subventricular zone (VZ/SVZ). The progenitors for cortically generated CalR^+^ subpopulation in primates are not yet characterized. Hence, the aim of this study was to identify patterns of expression of the transcription factors (TFs) that commit cortical stem cells to the CalR fate, with a focus on Gsx2. First, we studied the expression of Gsx2 and its downstream effectors, Ascl1 and Sp8 in the cortical regions of the fetal human forebrain at midgestation. Next, we established that a subpopulation of cells expressing these TFs are proliferating in the cortical SVZ, and can be co-labeled with CalR. The presence and proliferation of Gsx2^+^ cells, not only in the ventral telencephalon (GE) as previously reported, but also in the cerebral cortex suggests cortical origin of a subpopulation of CalR^+^ neurons in humans. *In vitro* treatment of human cortical progenitors with Sonic hedgehog (Shh), an important morphogen in the specification of interneurons, decreased levels of Ascl1 and Sp8 proteins, but did not affect Gsx2 levels. Taken together, our *ex-vivo* and *in vitro* results on human fetal brain suggest complex endogenous and exogenous regulation of TFs implied in the specification of different subtypes of CalR^+^ cortical interneurons.

## INTRODUCTION

The increased complexity of cortical progenitors is considered to be an evolutionary adaptation necessary for the development of higher brain functions in primates, and particularly in humans. Complex structure and function of the cerebral cortex critically depend on the balance of excitation and inhibition provided by pyramidal neurons and γ-aminobutyric acid-containing (GABAergic) interneurons, respectively. Interneurons comprise 20% of all cortical neurons in rodents and up to 35% in humans (Gabbott et al., 1997; DeFelipe et al., 2002; Jones, 2009). Importantly, their impairment has been described in various psychiatric and neurological disorders, including epilepsy, schizophrenia and autism (Yan et al., 1995; DeFelipe, 1999; Ulfig, 2001; Brandt et al., 2003; Baraban and Tallent, 2004; Lewis et al., 2005; Marin, 2012). In contrast to rodents, where the majority, if not all, cortical interneurons are generated in the ventral telencephalon (ganglionic eminence, GE; Anderson et al., 1997; Tamamaki et al., 1997; Parnavelas, 2000; Marin and Rubenstein, 2001), several groups have reported that cortical interneurons in primates originate both ventrally (in the GE) and dorsally, in the cortical subventricular zone (SVZ; Letinic et al., 2002; Rakic and Zecevic, 2003; Petanjek et al., 2009b; Jakovcevski et al., 2011; Al-Jaberi et al., 2013). This topic is still open for discussion since other groups reported that similar to rodents, the majority of cortical interneurons in primates originate in the GE and no proliferation of interneuron progenitors was demonstrated in the cortex (Hansen et al., 2013; Ma et al., 2013).

Cortical interneurons represent a heterogeneous group of cells that can be classified according to physiological, morphological or molecular criteria (Petilla Interneuron Nomenclature Group1 et al., 2008). In this study, we have focused on calretinin-expressing (CalR^+^) interneurons which have variable morphology and are comprised of bipolar and double bouquet cells (DeFelipe, 1997). In humans, up to 50% of all CalR^+^ cells are bipolar, localized predominantly in the upper cortical layers vs. approximately 15% in rodents (Condé et al., 1994; Yan et al., 1996; Gabbott et al., 1997; DeFelipe et al., 2006; Bayatti et al., 2008). By their electrophysiological output, they belong to accommodating or irregular-spiking interneurons (Porter et al., 1998) that target not only the distal dendrites of pyramidal cells, but importantly also other GABAergic interneurons. Their function in the primate cortex is complex, since they can disinhibit pyramidal cells by making synapses on each other (DeFelipe et al., 1999; Schlösser et al., 1999; Lewis et al., 2005; Zaitsev et al., 2005). The specific distribution of CalR^+^ cells in human upper cortical layers (II/III) suggests a role in cortical circuit formation necessary for higher brain functions specific to humans, such as abstract thinking and language (Hill and Walsh, 2005; Jones, 2009; Rakic, 2009).

Timing and specification of cortical interneuron development are guided by a variety of transcription factors (TFs). In mice, CalR^+^ interneurons originate mainly from the caudal ganglionic eminence (CGE; Xu et al., 2004; Butt et al., 2005) from progenitor cells expressing the TF genomic screened homeobox 2 (Gsx2; Hsieh-Li et al., 1995). The TFs Achaete-scute homolog 1 (Ascl1, originally named Mash1) and specificity protein 8 (Sp8) are downstream effectors of Gsx2 (Waclaw et al., 2009; Wang et al., 2009). Gsx2 has a role in the specification of cortical interneurons (Xu et al., 2010), olfactory bulb interneurons and striatal projection neurons (Stenman et al., 2003). In the human fetal telencephalon Gsx2 has been reported exclusively in the GE (Ma et al., 2013). However, its role in the generation of CalR^+^ interneurons in the developing human cerebral cortex still remains elusive.

It has been reported that CalR^+^ cells during early human developmental stages (from 6 gestational weeks, gw) are generated in the GE and tangentially migrate into the cortex (Zecevic et al., 1999; Meyer et al., 2000; Rakic and Zecevic, 2003), whereas by midgestation (20 gw) an additional subgroup of proliferating cells in the outer subventricular zone (oSVZ) express CalR, suggesting their local origin at that stage of development (Jakovcevski et al., 2011; Zecevic et al., 2011). Our *in vitro* studies confirmed that CalR^+^ cells originate from genetically labeled cortical human progenitors (Mo et al., 2007). Moreover, we reported that cortical radial glia cells (RGCs) *in vitro* generate CalR^+^ cells in human but not in mice (Yu and Zecevic, 2011).

Here, we describe a specific pattern of expression in the human fetal cortex of the three TFs proposed to be involved in the CalR lineage: Gsx2, and its downstream targets Ascl1 and Sp8. In addition, cortical RGCs cultures treated with Sonic hedgehog (Shh), a morphogen known to play a role in interneuron specification, show a selective effect on these TFs that may influence specification of CalR^+^ cells in human. These results indicate a complex endogenous and exogenous regulation of TFs implicated in the specification of the human CalR^+^ cortical interneurons.

## MATERIALS AND METHODS

### HUMAN TISSUE PROCESSING AND IMMUNOSTAINING

Fetal brain tissues at midgestation (14–24 gw, *n* = 22; **Table 1**) were obtained from the Tissue Repository at The Albert Einstein College of Medicine, Bronx, NY, USA and StemExpress, Diamond Springs, CA, USA. Handling of the human material was done with special care following all necessary requirements and regulations set by the Institutional Ethics Committee. Ultrasonography and gross neuropathological examination confirmed that the brain tissue was normal. Embryonic brains were fixed overnight in 4% PFA/0.1 M PBS (pH 7.4), then cryoprotected in 30% sucrose/PBS, frozen in TissueTek OCT and sectioned on a cryostat (15 μm). Slices were incubated for 1 h at room temperature (RT) in blocking solution (10% NGS and 0.5% Tween-20 in PBS). Primary antibodies (**Table 2**) were applied overnight at 4°C in blocking solution followed by corresponding secondary antibodies (Jackson Immuno-Research Lab, West Grove, PA, USA) for 1 h at RT, and a short incubation in a nuclear stain bis-benzimide (Sigma).

**Table 1:** Fetal human brain tissues analyzed in the study.

Cases	Gestational week (gw)	Gender	Direct tissue application	Cell culture application
1	14	NP	–	WB
2	15	♀	IHC	–
3	16	♂	RT-PCR	–
4	16	♀	IHC	–
5	17	♂	–	RT-PCR, WB
6	17	NP	IHC	–
7	17	♂	IHC	–
8	17	♂	–	WB
9	18	NP	RT-PCR	RT-PCR, WB
10	18	♂	IHC/ISH	–
11	18	NP	IHC	–
12	19	NP	–	WB
13	19	♀	IHC	–
14	20	♀	IHC/ISH	–
15	20	NP	IHC/ISH	–
16	21	NP	IHC/ISH	–
17	21	NP	IHC/ISH	–
18	22	NP	IHC/ISH	–
19	22	♂	IHC/ISH	–
20	23	♀	IHC/ISH	–
21	24	♂	IHC/ISH	–
22	24	♀	IHC/ISH	–

*♀, female; ♂, male; ICC, Immunocytochemistry; IHC, Immunohistochemistry; ISH, *In situ* hybridization; NP, Not provided; RT-PCR, real-time PCR; WB, Western blot*

**Table 2:** Primary antibodies used in this study (in alphabetical order).

Antigen	Host	Clone	Dilution	Manufacturer	Catalog no.
Ascl1	Mouse IgG1	24B72D11.1	1:500	BD Pharmingen	556604
β-Actin	Mouse IgG		1:2000	Thermo Scientific	MA5-15739
CalR	Mouse IgG Rabbit IgG	6B8.2	1:500 1:500	Millipore Chemicon Sigma	MAB1568 PA007306
GABA	Rabbit IgG		1:2000	Sigma	A2052
Gsx2	Rabbit IgG Mouse IgG		1:250 1:500	Abcam Millipore Chemicon	AB26255 ABN162
Ki67	Mouse	MIB1	1:50	DAKO	M7240
Nkx2.1	Rabbit IgG	EP1584Y	1:500	Abcam	AB76013
Sp8 (C-18)	Goat IgG		1:500	Santa Cruz	Sc-104661
Sox2	Goat IgG		1:500	Santa Cruz	Sc-17320
Tbr1	Rabbit IgG		1:500	Proteintech	20932-1-AP
Tbr2	Rabbit IgG		1:500	Gift from R. Hevner	

### *IN SITU* HYBRIDIZATION

The human *Gsx2* full coding sequence plasmid was obtained from OpenBiosystems (IMAGE:30915601). Riboprobe was generated from the linearized vector construct by *in vitro* transcription using digoxigenin (DIG)-UTP (Roche) as the label. *In situ* hybridization (ISH) was performed on cryosections (15 μm) described above. Sections were dried at RT for 2 h and subsequently fixed for 10 min with 4% PFA, before overnight incubation at 68°C in hybridization buffer 1× DEPC-treated “salts” (200 mM NaCl, 5 mM EDTA, 10 mM Tris, pH 7.5, 5 mM NaH_2_PO_4_⋅2H_2_O, 5 mM Na_2_HPO_4_; Sigma–Aldrich), 50% deionized formamide (Roche), 0.1 mg/ml RNase-free yeast tRNA (Invitrogen), 1× Denhardts (RNase/DNase free; Invitrogen), 10% dextran sulfate (Sigma-Aldrich) containing 100–500 ng/ml DIG-labeled RNA probe. After hybridization, sections were washed three times in a solution containing 50% formamide 1× SSC (Invitrogen) and 0.1% Tween 20 (Sigma–Aldrich) at 65°C, and two times at RT in 1× MABT (20 mM maleic acid, 30 mM NaCl, 0.1% Tween 20; Sigma-Aldrich) before incubating in a solution containing 2% blocking reagent (Roche) and 10% heat inactivated sheep serum (HISS) in MABT, followed by overnight incubation in alkaline phosphatase (AP)-conjugated anti-DIG antibody (1:1500; Roche Applied Science). Fast Red (Roche) was used for fluorescent color detection of probe (FISH) by incubation in 100 mM Tris, pH 8.2, 400 mM NaCl containing Fast Red for 1–2 h at 37°C. Sections were counterstained with bis-benzimide and coverslipped using Fluoromount G mounting medium. Specificity of the procedure was assessed with a probe corresponding to the sense strand of *Gsx2*.

### IMMUNOHISTOCHEMISTRY AFTER *IN SITU*

Following overnight incubation with mouse anti-Ascl1, rabbit anti-calretinin and goat anti-Sp8 antibodies, sections were thoroughly washed in PBST (0.2% Triton) and incubated with Alexa 488 secondary antibody to detect immunoreactivity. Nuclei were counterstained with bis-benzimide.

### DISSOCIATED MIXED CELL CULTURE AND ENRICHMENT OF RGCs

Human fetal brain tissue (*n* = 5) ranging in age from 14 to 19 gw was obtained from Advanced Bioscience Resources (ABR, Alameda, CA, USA) and StemEx (Diamond Springs, CA, USA) with proper parental consent and the approval of the Ethics Committees. Brain tissue was collected in oxygenized Hank’s balanced salt solution (HBSS; LifeTechnologies, Grand Island, NY, USA) and transported on ice. Dissociated cell cultures were prepared from dorsal and ventral regions of the telencephalon as described previously (Zecevic et al., 2005). Isolated tissue of interest was mechanically dissociated and enzymatically degraded at 37°C for 30 min with 0.025% trypsin (Gibco). Afterward, DNase (Sigma Aldrich, St. Louis, MO, USA; 2 mg/ml) was added to the cell suspension and cells were washed in HBSS (LifeTechnologies, Grand Island, NY, USA). Cells were resuspended in the proliferation medium consisting of DMEM/F12 (LifeTechnologies) with 10 ng/ml basic fibroblast growth factor (bFGF; Peprotech, Rocky Hill, NJ, USA), 10 ng/ml epidermal growth factor (EGF Millipore, Billerica, MA, USA) and supplemented with B27 (LifeTechnologies). Cells were kept in the proliferating medium 7–10 days until 80% confluence was achieved. A surface marker CD15 (Lex) was used for immunomagnetic cell sorting of RGCs using MACS columns (Miltenyi Biotec, Auburn, CA, USA) which resulted in 96% purity of RGCs (Mo et al., 2007; Yu and Zecevic, 2011). For immunocytochemistry approximately 250,000 cells were plated on coverslips coated with poly-D-lysine (Sigma–Aldrich). For total protein and RNA isolation approximately 2 million cells were plated in poly-D-lysine coated wells. In order to confirm the identity of isolated cells, 24 h after isolation, live immunocytochemistry was performed using markers for radial glia, CD15 and brain lipid binding protein (BLBP). After 3 days *in vitro* (DIV), cells were transferred from proliferation to differentiation medium (DM; DMEM/F12/B-27 without bFGF and EGF) and kept for an additional 7 DIV.

### TREATMENT OF CELL CULTURES AND IMMUNOCYTOCHEMISTRY

Cells were treated for 7 DIV (every third day) in DM with a combination of recombinant human Shh (C24II), N-terminus (200 ng/ml; R&D systems, Minneapolis, MN, USA) and purmorphamine (PMM; 1 μM; Calbiochem Millipore), an agonist of Smoothened receptor and cyclopamine, an antagonist of Smo receptor (2.5 μM; EnzoLife Sciences, NY, USA). Control cells were kept in DM. Cells were fixed in 4% paraformaldehyde 24 h after isolation of RGCs and 7 DIV after pharmacological treatment. Primary antibodies diluted in blocking solution (1% bovine serum albumin, 5% normal goat serum, and 0.5% Tween-20 in PBS) were applied overnight at +4°C, followed by corresponding secondary Alexa 488- or Alexa 555- conjugated antibodies (Life Technologies) for 2 h at RT. Nuclei were counterstained with the nuclear stain bis-benzimide. Cells were visualized with a Zeiss fluorescence microscope using Axiovision software and photographed with a digital camera. Ten pre-designated adjacent optical fields of view were examined at magnification 10× (0.5 mm^2^ surface area); counts of immunolabeled cells were pooled together, expressed as means ± SEMs (Standard Error of the Means) and analyzed using Student’s *t*-test. The criterion for significance was set at 5%.

### WESTERN BLOT

Cells were homogenized in lysis buffer (50 mM Tris-HCl pH 7.4, 150 mM NaCl, 1% NP-40, 1 mM phenylmethylsulphonyl fluoride, and protease inhibitor cocktail) on ice for 30 min, centrifuged at 14,000*g* for 15 min at 4°C, and the supernatants were collected as the cell lysates. Equal amounts of protein from each sample were separated by SDS-PAGE on 12% gels and transferred to nitrocellulose membranes (Bio-Rad, Hercules, CA, USA). Used primary antibodies are listed in **Table 2**. Membranes were incubated with the primary antibodies overnight at 4°C, and then with their corresponding secondary HRP-conjugated antibodies (1:15000, Thermo Fisher Scientific, Temecula, CA, USA). Protein signal was detected using SuperSignal West Pico Chemiluminescent system (Thermo Fisher Scientific, Temecula, CA, USA). Western blots were scanned and densitometric analysis was performed with ImageJ software (National Institutes of Health, Bethesda, MD, USA). Statistical analyses were performed using paired *t*-test. The criterion for significance was set at 5%.

### REAL-TIME PCR

Real-time PCR (RT-PCR) was used to determine mRNA expression of GAPDH, Gsx2, Ascl1 and Sp8. Total RNA was extracted from cells using TRIZOL®; reagent (Invitrogen, Carlsbad, CA, USA) according to the manufacturer’s instructions. Approximately 1 μg of RNA was used in the reverse transcription reaction using M-MuLV reverse transcriptase with random hexamers (Fermentas, Vilnius, Lithuania) according to the manufacturer’s instructions. RT-PCR was performed in a Realplex^2^ Mastercycler (Eppendorf, Hamburg, Germany) using 96-well reaction plates (Eppendorf, Hamburg, Germany). The reactions were prepared according to the standard protocol for one-step QuantiTect SYBR Green RT-PCR (Applied Biosystems, Cheshire, UK). The sequences of the forward and reverse primers are presented at **Table 3**. The thermal cycle conditions were 95°C for 2 min followed by 40 cycles of 15 s at 95°C, 15 s at 55°C, and 20 s at 68°C. All assays were performed in triplicates. Averaged cycle of threshold (Ct) values of GAPDH triplicates were subtracted from Ct values of target genes to obtain ΔCt, and then relative gene expression was determined as 2^-ΔCt^. The results were presented relative to the control value, which was arbitrarily set to 1.

**Table 3:** List of primer sequences used in the study.

Gene	Forward	Reverse
Ascl1(Mash1)	TCTCATCCTACTCGTCGGACGA	CTGCTTCCAAAGTCCATTCGCAC
GAPDH	ACCACCATGGAGAAGGC	GGCATGGACTGTGGTCATGA
Gsx2	GGAGATTCCACTGCCTCACCAT	CGGAGTCGAGACAGGTACATGT
Sp8	GAGGCTACAACTCGGATTACTCG	GTAGCACTGGCTTGAAGCCGTC

## RESULTS

### Gsx2 IS EXPRESSED IN THE HUMAN DEVELOPING CEREBRAL CORTEX

**FIGURE 1:**
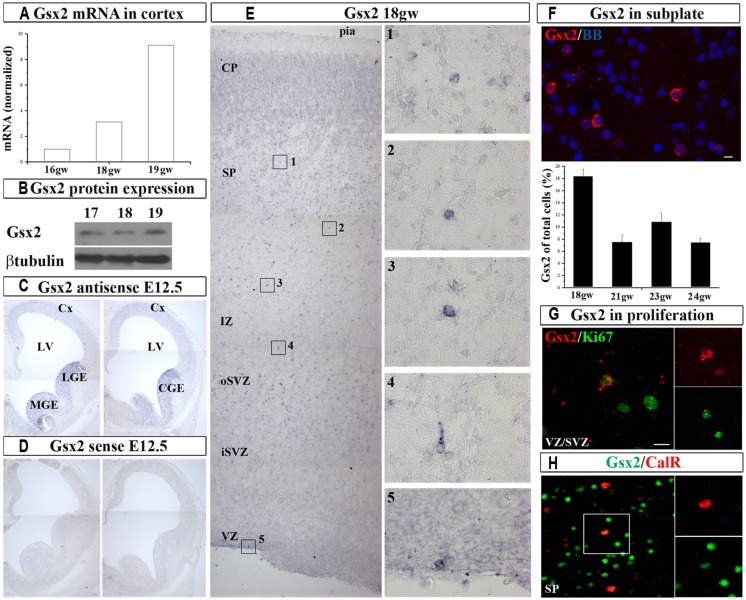
**Gsx2 in the human and mouse developing brain at mid-gestation.** Expression of Gsx2 mRNA **(A)** and protein **(B)** in the human fetal cortex. *In situ* hybridization in E12.5 mouse with Gsx2 anti-sense **(C)** and sense **(D)** probe. **(E)** ISH in human fetal cerebral cortex (18 gw); insets- high magnification of Gsx2^+^ cells; **(F)** Representative Gsx2^+^ cells in subplate (FISH) for quantification (23 gw); percentage of Gsx2^+^ cells from total cells is shown in the histogram in the different stages of development (18–24 gw). **(G)** *In situ* signal for Gsx2^+^ cells (red) co-labeled with anti-Ki67 antibody (green) in cortical VZ (optical sectioning); **(H)** CalR^+^ cells (red) in subplate colabeled with Gsx2 (green; 18 gw). VZ, ventricular zone; iSVZ, inner subventricular zone; oSVZ, outer subventricular zone; IZ, intermediate zone; SP, subplate; CP, cortical plate; Cx, cortex; LV, lateral ventricle; CGE, caudal ganglionic eminence; MGE, medial ganglionic eminence. Scale bars 10 μm.

In order to establish if Gsx2 is present in the cortex of human developing telencephalon at midgestation, we dissected fresh dorsal (cortical; 16–19 gw) and ventral (GE) telencephalon (16–18 gw) and determined Gsx2 mRNA expression levels (**Figure 1A**). RT-PCR demonstrated that Gsx2 mRNA levels in the GE at 16 and 18 gw were 345 folds and 104 folds higher respectively compared to the cortex. Notably mRNA Gsx2 was present in the cortical tissue in all investigated samples (**Figure 1A**). An increase of the cortical Gsx2 mRNA levels was observed during development, changing three to nine folds from 16 to 18 and 19 gw, respectively (**Figure 1A**). Protein levels for Gsx2 in fetal cerebral cortex at 17–19 gw followed the trend seen with mRNA (**Figure 1B**). Thus, in addition to regional differences, Gsx2 expression in the human fetal brain seems to vary with gestational age, although this point needs to be confirmed on more cases.

Next, we sought to determine the expression pattern of Gsx2 in human fetal brain and compare it with the mouse using ISH. At E12.5 mouse brain characteristic Gsx2 expression was detected in the medial (MGE), lateral (LGE), and CGE but not in the pallium, in agreement to previous reports (Hsieh-Li et al., 1995; **Figures 1C,D**). However, in the human fetal cerebral cortex Gsx2 transcript was observed in the cortical ventricular/subventricular zone (VZ/SVZ), intermediate zone (IZ), subplate (SP), and cortical plate (CP) regions in all studied ages, from 18 to 24 gw (*n* = 10, **Figures 1E,F**). At this developmental stage, the highest expression was observed in the SP (**Figure 1F**). Occasionally Gsx2^+^ cells were also observed on the ventricular surface which suggested their proliferation (**Figure 1E**). To explore this possibility, we performed fluorescent *in situ* hybridization (FISH) for Gsx2 combined with immunoreaction to anti-Ki67 antibody, which labels cycling cells. We estimated that 20% of all Gsx2^+^ cells in the VZ/SVZ were proliferating as they were co-labeled with Ki67. Hence, not only that Gsx2^+^ cells were present in the human developing cortex at midgestation, but a fraction of these cells were proliferating locally. Our findings differ from previously published reports where Gsx2^+^ cells have been exclusively described in the subpallium in both rodents (Corbin et al., 2003; Xu et al., 2010) and humans (Ma et al., 2013). The observed difference in these studies could be due to the different methodological approaches followed. Here, in addition to immunolabeling with commercially available antibodies, we performed ISH, a high stringency conditioned method, to detect a specific signal for Gsx2 mRNA in the cortex.

After demonstrating the expression of Gsx2 in the human developing cortex, we stained cryosections with anti-Gsx2 and anti-CalR antibodies and quantified the number of co-labeled cells. Immunolabeling of Gsx2^+^ cells confirmed ISH results, with the highest number of cells observed in the SP and CP, accounting for about 10% of all cells in those regions at 17–19 gw (*n* = 8). Importantly, approximately one-third of all CalR^+^ cells during midgestation were co-labeled with Gsx2 (30% at 15–16 gw, 29% at 17–19 gw; **Figure 1H**), suggesting that Gsx2 expression is important for the CalR lineage during this time of development and in this cortical region.

### DISTRIBUTION OF Ascl1 AND Sp8 IN THE HUMAN DEVELOPING FOREBRAIN

**FIGURE 2:**
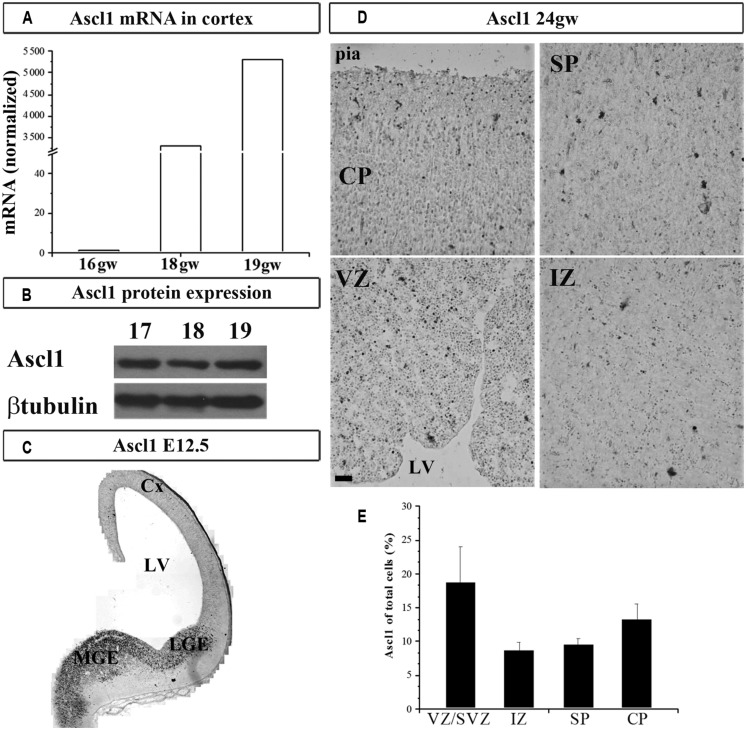
**Ascl1 in the human and mouse developing brain at mid-gestation.** Expression of Ascl1 mRNA **(A)** and protein **(B)** in the human fetal cortex. Immunohistochemistry in E12.5 mouse with anti-Ascl1 antibody **(C)**. **(D)** Representative immunolabeled Ascl1^+^ in human fetal cerebral cortex (24 gw). **(E)** Percentage of Ascl1^+^ cells in all nuclei (labeled with bis-benzimide) in the human fetal neocortex at midgestation (21–24 gw). Data are presented as mean values + standard error of the mean (minimum three sections from two brains were studied). VZ/SVZ, ventricular/subventricular zone; IZ, intermediate zone; SP, subplate; CP, cortical plate; Cx, cortex; LV, lateral ventricle; CGE, caudal ganglionic eminence; MGE, medial ganglionic eminence. Scale bar 50 μm.

We next studied the expression of TF Ascl1 in the human cortex, a reported downstream target of Gsx2 in rodents. We detected Ascl1 mRNA in fetal cortical VZ/SVZ (**Figure 2A**), which confirmed previous results obtained by immunolabeling in fetal human (Letinic et al., 2002; Jakovcevski et al., 2011) and non-human primate brains (Petanjek et al., 2009a). Expression of Ascl1 was higher in the GE compared to the cortex: 22 and 5-folds at 16 and 18 gw, respectively. Similar to our findings on Gsx2, the expression levels of Ascl1 mRNA increased in the course of development, from 16 to 18 gw (**Figure 2A**). Ascl1 expression was also demonstrated at the protein level (**Figure 2B**). To compare our results to mouse, we immunolabeled Ascl1 in mouse developing brain (E12.5) and observed the presence of Ascl1^+^ cells mainly in the subpallium (**Figure 2C**). We next performed a similar experiment in the human forebrain at 24 gw, and showed that, besides the strong immunolabeling of the GE previously shown (Jakovcevski et al., 2011; Hansen et al., 2013), considerable immunoreactivity was present in the cortical regions (**Figure 2D**). The density of Ascl1^+^ cells in the human cortex at midgestation was the highest in the VZ/SVZ where it reached almost 20% of all nuclei, followed by around 14% in the CP and 10% in the IZ and SP (**Figure 2E**). Notably, in the CP Ascl1^+^ cells were preferentially distributed in the upper cortical layers (II/III; **Figure 2D**). We previously co-labeled cells with either CalR or GABA and Ascl1 antibodies and found that in the CP up to 50% of Ascl1^+^ cells could be co-labeled with GABA (Jakovcevski et al., 2011).

**FIGURE 3:**
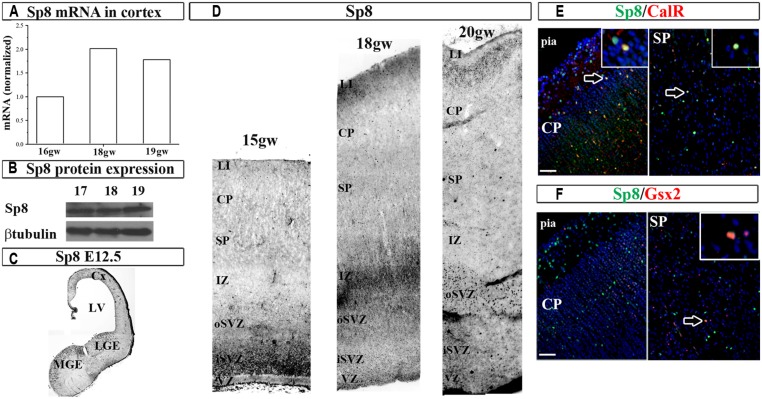
**Sp8 in the human and mouse developing brain at mid-gestation.** Expression of Sp8 mRNA **(A)** and protein **(B)** in the human fetal cortex. Immunohistochemistry in E12.5 mouse with anti-Sp8 antibody **(C)**. **(D)** Representative immunolabeled Sp8^+^ cells in the human fetal cerebral cortex (15–20 gw). Sp8^+^ cells (green) co-labeled with **(E)** CalR (red) and **(F)** Gsx2 (red) in cortical plate and subplate. VZ, ventricular zone; iSVZ, inner subventricular zone; oSVZ, outer subventricular zone; IZ, intermediate zone; SP, subplate; CP, cortical plate; Cx, cortex; LV, lateral ventricle; CGE, caudal ganglionic eminence; MGE, medial ganglionic eminence. Scale bar, 50 μm.

Next, we studied the expression of the zinc finger TF Sp8 required for the normal development of CalR interneurons in the olfactory bulb (Waclaw et al., 2006). In the human fetal cortex immunoreactivity for Sp8 has been observed in the dorsal LGE (dLGE) and dCGE, and a weak expression in the cortical VZ/SVZ (Ma et al., 2013). In addition, the same group reported that some migrating neurons in the CP were co-labeled with Sp8, GABA and chicken ovalbumin upstream promoter TF II (CoupTFII) antibodies, indicating their CGE origin (Ma et al., 2013). In the current study we extended these results by demonstrating not only Sp8 protein, but also the Sp8 mRNA expression in human fetal cortical tissue (**Figures 3A,B**). In the two cases studied (16 and 18 gw) Sp8 mRNA was higher in the GE than in cortex six and twofolds, respectively, and as previously observed with Gsx2 and Ascl1, cortical levels increased approximately two times from 16 to 18 and 19 gw (**Figure 3A**). In comparison to the E12.5 mouse brain where Sp8^+^ cells were revealed in the MGE, LGE and cortex as reported previously (Sahara et al., 2007; Borello et al., 2013; **Figure 3C**), in human, Sp8^+^ cells were also packed in the cortical SVZ/VZ and scattered in SP and CP (**Figure 3D**). Double-labeling experiments have shown that subpopulations of cortical Sp8^+^ cells co-expresses CalR or Gsx2, suggesting that a subpopulation of them are cortical interneurons (**Figures 3E,F**). However, further studies are needed to establish a direct lineage relationship of Gsx2 to CalR^+^ cells in the human cortex.

### CORTICAL AND GE HUMAN RGCs HAVE DIFFERENT POTENTIAL TO GENERATE CalR^+^ CELLS

Previously, we have shown that cortical RGCs *in vitro* have the potential to generate CalR^+^/GABA^+^ interneurons and Nkx2.1^+^ progenitors (Yu and Zecevic, 2011; Radonjic et al., in press). Here, we explore if cortical and GE RGCs retain their expression of regional characteristic markers *in vitro* and compare their capacity to produce GABA and CalR^+^ cortical interneurons.

**FIGURE 4:**
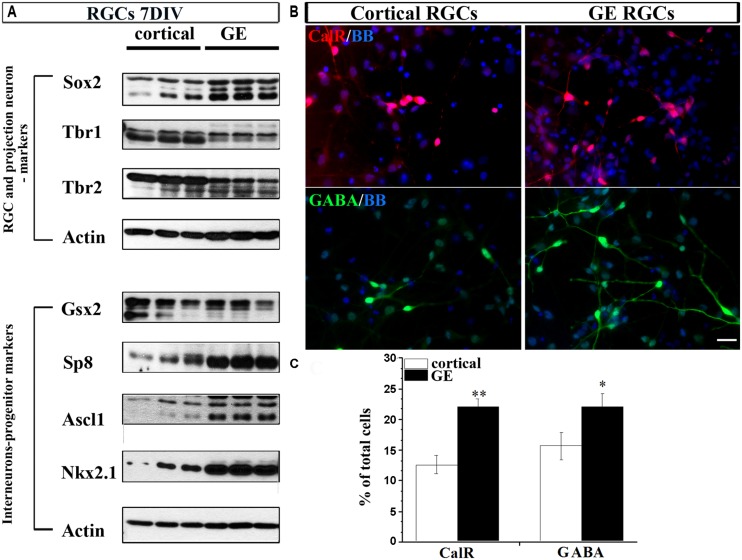
**Differentiation of RGCs enriched from cortical or GE regions. (A)** Markers of RGCs, projection neurons and interneurons expressed in cortical and GE enriched RGCs after 7 DIV. **(B)** Representative staining of CalR^+^ (red) and GABA^+^ (green) cells after 7 DIV in cortical and GE RGCs. **(C)** Histogram shows the percentage of CalR^+^ and GABA^+^ cells from the total cells differentiated after 7 DIV in cortical and GE RGC cultures. *p < 0.05, **p < 0.01; *t*-test. Scale bar, 20 μm.

To this end, we dissected cortical VZ/SVZ and ventral telencephalon (GE) and established dissociated cell cultures. To isolate RGCs, we immuno-sorted CD15^+^ cells, a specific surface marker of RGCs (Mo et al., 2007; Yu and Zecevic, 2011; Ortega et al., 2013). Cortical and GE RGC cultures were differentiated for 7 DIV and the expression of characteristic markers for RGCs (Sox2), projection neurons (Tbr1 and Tbr2) and interneuronal progenitors (Gsx2, Sp8, Ascl1, and Nkx2.1) were analyzed. Expression of typical markers of glutamatergic neurons Tbr1 and Tbr2 was higher in cortical RGC cultures than in GE RGC cultures (**Figure 4A**). In contrast, the expression of Sp8, Ascl1, and Nkx2.1 was higher in GE RGC cultures (**Figure 4A**), but no difference in Gsx2 expression was observed in these two regions. After 7 DIV both cortical and GE RGCs generated CalR^+^ and GABA^+^ cells, but with a different capacity. Namely, more CalR^+^ (22 vs.12%) and GABA^+^ (22 vs.16%) cells were generated in the GE RGC cultures than in dorsal cultures (**Figures 4B,C**). These results are in agreement with our previous reports (Mo et al., 2007; Mo and Zecevic, 2008). Hence, we concluded that cortical and GE RGC cultures retain *in vitro* regional identities as well as the potential to generate GABAergic interneurons.

### EXOGENOUS Shh AFFECTS THE EXPRESSION OF TRANSCRIPTION FACTORS UPSTREAM OF CalR

The morphogen Shh has numerous functions in the developing central nervous system (Dahmane and Ruiz-i-Altaba, 1999). One of function is the induction of the ventral TFs Nkx2.1 (thyroid transcription factor-1, TTF1), Dlx1/2 and Ascl1 and specification of cortical interneurons in the ventral telencephalon of rodents (Anderson et al., 1997; Kohtz et al., 1998; Sussel et al., 1999; Xu et al., 2004, 2005; Butt et al., 2005). In the human fetal cerebral cortex, we previously observed Shh expression in the Map2^+^ neurons in the fetal CP/SP and Sox2^+^ RGCs in the VZ (Radonjic et al., in press). Treatment of cortical RGC cultures with exogenous Shh suppressed the generation of CalR^+^ interneurons in favor of Nkx2.1^+^ progenitors, suggesting that in humans Shh differentially affects subgroups of cortical progenitors (Radonjic et al., in press).

**FIGURE 5:**
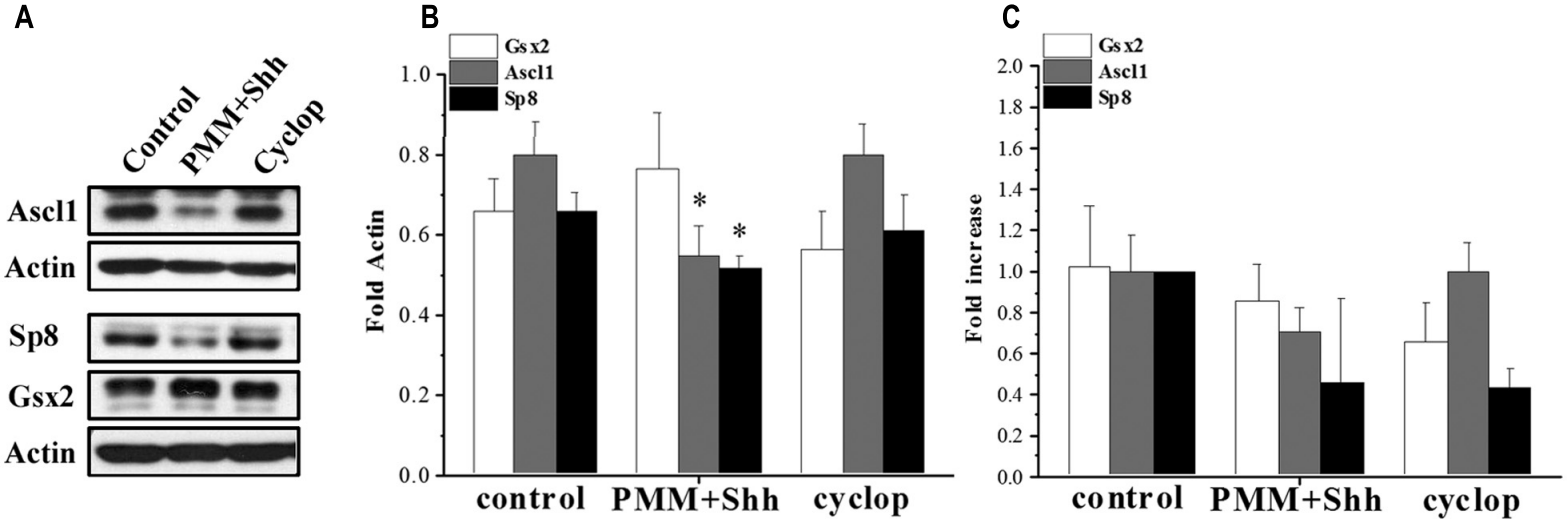
**Effect of Shh signaling on Gsx2, Ascl1, and Sp8 in cortical RGC cultures after 7 DIV. (A)** Representative immunoblots and **(B)** quantification on the protein expression and **(C)** mRNA levels after treatment with PMM/Shh and cyclopamine. *p < 0.05, *t*-test.

Here, using *in vitro* enriched RGCs we examined the effect of Shh on TFs upstream of CalR^+^ cells and demonstrated that treatment of cortical RGCs with PMM/Shh reduced protein levels of Ascl1 and Sp8, but not Gsx2 levels (**Figure 5**). However, treatment with cyclopamine, an inhibitor of Shh signaling, did not affect expression levels of these TFs (**Figures 5A,B**). The lack of effect of cyclopamine suggests that additional Shh independent pathways might control cortical interneurongenesis, as it is a case with oligodendrocyte progenitors (Nery et al., 2001; Ortega et al., 2013) and Nkx2.1^+^ progenitors (Radonjic et al., in press). The mRNAs levels of Gsx2, Ascl1 and Sp8 obtained by PCR analysis showed similar trend, but the differences did not reach significance (**Figure 5C**). Although additional experiments are needed to formulate final conclusions, these results suggest that in human cortical development Shh could differentially modulate distinct TFs implicated in the generation of CalR^+^ interneurons.

## DISCUSSION

The potential origin and progenitors of CalR^+^ interneurons in the human cerebral cortex are still under debate. Here, we demonstrated the expression pattern of three TFs, Gsx2 and its downstream effectors Ascl1 and Sp8, both in the GE and in the cortex of the fetal human forebrain at midgestation. A fraction of cells expressing these TFs were co-labeled with CalR, suggesting their lineage relationship. Notably, we identified a potentially important species difference in the expression of Gsx2. While in the mouse cortex, Gsx2 mRNA was not observed, human fetal cortical cells express this transcript and importantly a subset of these cells was proliferating in the cortical VZ/SVZ. Thus, our results support the view that both cortical and GE regions of the human fetal telencephalon have the capacity to give rise to cortical CalR^+^ cells. Finally, we show that the mechanism underlying the effects of Shh on the reduction of CalR interneurons involves the down-regulation of Ascl1 and Sp8 expression.

### TRANSCRIPTION MECHANISMS INVOLVED IN THE GENERATION OF CalR CELLS

In mice, bipolar CalR^+^ interneurons originate mainly from Gsx2^+^ germinal zones of LGE and CGE (Fogarty et al., 2007; Sahara et al., 2007; Miyoshi et al., 2010; Xu et al., 2010). It is estimated that the CGE gives rise to approximately 30–40% of all cortical interneurons (Grateron et al., 2003; Miyoshi et al., 2010). Use of inducible genetic fate mapping techniques demonstrated that 75% of labeled precursors from the CGE contribute to the superficial cortical layers regardless of their birth date (Miyoshi et al., 2010). A required downstream effector of Gsx2 is Ascl1 (Wang et al., 2009). Both Gsx2^+^ and Ascl1^+^ neural progenitor cells express CoupTFII (Kanatani et al., 2008), a TF that labels cells in the CGE (Yozu et al., 2005; Kanatani et al., 2008; Miyoshi et al., 2010). In human cortex at mid-gestation 60–75% of CalR^+^ cells, depending on the cortical layer, co-express CoupTFII (Reinchisi et al., 2012). Other studies in human and monkey fetal brains found that nearly all cortical CalR^+^ cells co-express either CoupTFII or/and Sp8 and thus originated in the CGE and dLGE (Hansen et al., 2013; Ma et al., 2013). Another downstream effector of Gsx2 is Sp8, a TF that plays a role in cortical patterning, proliferation and differentiation of cortical progenitors (O’Leary and Sahara, 2008; Waclaw et al., 2009; Wang et al., 2009; Cai et al., 2013). In rodents, Sp8 is present in 20% of cortical interneurons that originate from dLGE and CGE and express Reelin, VIP, NPY, and CalR (Ma et al., 2012). Similar to rodents, in the human fetal telencephalon Sp8 immunoreactivity was demonstrated in the dLGE and CGE, and in postmitotic neurons in the cortex (Ma et al., 2013; Hansen et al., 2013). Our results show that at midgestation cortical Sp8^+^ cells were mainly localized to the SVZ/VZ, but also in upper cortical regions. Notably, a subpopulations of cortical Sp8^+^ cells co-express CalR or Gsx2, suggesting that they are cortical interneuron progenitors.

Although recent results point to the CGE as the major source of CalR^+^ cells, a fraction of proliferating CalR^+^ cells was observed in the human cortical VZ/SVZ at midgestation (Jakovcevski et al., 2011; Zecevic et al., 2011). Further corroboration that a subtype of CalR^+^ cells has cortical origin came from our result that 20% of Gsx2^+^ cells, presumably progenitors of CalR^+^ cells, are dividing in the cortical VZ/SVZ. Additionaly, both *in vivo* and *in vitro* approaches showed the potential of human cortical Pax6^+^/BLBP^+^ RGCs to produce CalR^+^ cells. We previously reported that in human fetal cortical VZ/SVZ, CalR is co-localized in a subpopulation of Pax6^+^ cells, and CalR^+^ cells can be produced from genetically labeled BLBP^+^ cortical RGCs *in vitro* (Mo and Zecevic, 2008; Yu and Zecevic, 2011). Moreover, knocking down Pax6 in cortical RGCs, greatly reduced the generation of CalR^+^ cells (Mo and Zecevic, 2008). Taken together, these results suggest an important role of Pax6 not only for genesis of glutamatergic neurons, but also CalR^+^ cells. However, we did not observe GABA in the Pax6^+^ progenitors, suggesting that GABA is expressed only in postmitotic cells that already down-regulated Pax6.

Thus, our results reinforced the observation that in primates a number of progenitors related to cortical interneurons have a mitotic origin in the cortical VZ/SVZ at midgestation. These include CalR^+^, Ascl1^+^, and Nkx2.1^+^ (Jakovcevski et al., 2011), CoupTFII^+^ (Reinchisi et al., 2012) and Gsx2^+^ cortical progenitors (this study). Other groups have also found that cortical cells expressing Ascl1 and co-labeled with interneuron markers proliferate in human and the non-human primate VZ/SVZ (Letinic et al., 2002; Petanjek et al., 2009b).

These findings are consistent with the idea that the cortical VZ/SVZ is an additional source of interneurons in the primate developing cortex. However, this issue is still under debate since other groups reported either no expression of those TFs (Gsx2) in cortical areas or the expression only in postmitotic cells (Sp8, CoupTFII, Nkx2.1; Hansen et al., 2013; Ma et al., 2013). These discrepancies might be methodological, due to the different tissue preparation, *post mortem* time or antibodies used. To minimize the inherent variability in human tissue sampling, we tried to confirm our results at both the mRNA and protein level using a combination of histological techniques (ISH and immunohistochemistry, respectively) as well as Western blot and RT-PCR analysis of the tissue homogenates. Due to scarce availability of human fetal brain tissue, it is not unusual that such discrepancies arise and we hope that further studies with larger samples and better standardized methods would resolve the still open questions about the origin of cortical interneurons in primates. We must emphasize, however, that the finding of interneuron progenitors in cortical regions of the primate brain does not preclude the possibility that some of these cells have originated in the GE, migrated as intermediate progenitors and continue to proliferate in the cortical VZ/SVZ, as already suggested in mice (Wu et al., 2011). Both mechanisms could be present simultaneously with a goal to increase the number of cortical interneurons in the enlarged primate cerebral cortex.

Notably, the cortical and GE VZ/SVZ are not the only sources of cortical interneurons in later stages of human brain development. Still another probable source is a primate-specific subpial granular layer (SGL), transiently present under the pia in humans (Brun, 1965; Gadisseux et al., 1992; Meyer and Wahle, 1999; Zecevic and Rakic, 2001) and monkey developing brains (Zecevic and Rakic, 2001). Various sources for CalR^+^ cells are probably necessary to supply increased upper cortical layers with higher numbers of CalR^+^ interneurons in primates compared to other species. Importantly, different origins of CalR^+^ cells are likely to contribute to the diversity of this subpopulation of interneurons in primates. The increased complexity of cortical progenitors is considered to be an evolutionary adaptation necessary for the development of higher brain functions in primates, and particularly in humans.

### EXTERNAL FACTORS AFFECTING CalR EXPRESSION

Clonal lineage studies of non-pyramidal neurons suggested that the expression of calcium binding proteins is not genetically programmed and is likely to be induced by functional activity (Alcántara et al., 1993; Grateron et al., 2003) and external factors (Mione et al., 1994). For example, bFGF stimulates the generation and differentiation of CalR^+^ neurons, and its effects are enhanced by retinoic acid (Pappas and Parnavelas, 1998). Another external factor affecting CalR^+^ cells in mice is Shh (Butt et al., 2005; Xu et al., 2005, 2010). Exogenous Shh treatment of MGE progenitors in mice resulted in a down-regulation of both CalR and Gsx2, whereas down-regulation of Shh in the MGE resulted in conversion of interneuron fate from PV and Sst^+^ to bipolar CalR^+^ (Xu et al., 2005, 2010; Carney et al., 2010). Human stem cells are used by many groups as a model for studying the development of human cortical interneurons (Mariani et al., 2012; Maroof et al., 2013). In our study, we used instead RGCs isolated from the human developing cortex to study the molecular mechanisms underlying interneuron generation and specification. We observed that cortical and GE RGCs *in vitro* retain different potential to generate interneurons. This result is in line with previous *in vitro* results demonstrating that enriched RGCs maintain their regional identity (Radonjic et al., in press). Treatment of cortical human RGCs resulted in down-regulation of Ascl1 and Sp8, while there was no effect on Gsx2 expression. We hypothesize that this decrease is either due to re-specification to Nkx2.1^+^ progenitors or maintenance of Gsx2^+^ progenitor state. We demonstrated recently that treatment with Shh reduced the number of CalR^+^ cells generated in RGC cultures (Radonjic et al., in press), thus it is tempting to speculate that Shh arrests further differentiation of Gsx2 progenitors into CalR^+^ cells.

### TRANSIENT CalR EXPRESSION IN CORTICAL PROGENITORS DURING DEVELOPMENT

In this study we describe the expression pattern of CalR^+^ cells during human brain development in presumably interneuron progenitors. However, the question remains if CalR expression during development is transient or stable. In the primate cortex, CalR^+^ cells can be detected already at 5 gw with a gradient from subcortical GE to the neocortex (Rakic and Zecevic, 2003; Zecevic et al., 2011). However, the earliest CalR^+^ cells in the preplate zone cannot be considered interneurons (Meyer et al., 2000; González-Gómez and Meyer, 2014). Later during development, at midgestation, CalR^+^ cells almost entirely overlap with immunoreactivity to GABA (Rakic and Zecevic, 2003; Zecevic et al., 2005). During development however, the number of CalR^+^ cells decreases while at the same time levels of other calcium binding proteins expressed in GABAergic neurons, such as calbindin or parvalbumin, increase (Yan et al., 1995, 1996; Schlösser et al., 1999; Brandt et al., 2003). In addition, the percentage of CalR^+^/GABA^+^ cells is reduced from 96% at midgestation to 37% in adulthood in primates (Del Río and DeFelipe, 1994; Yan et al., 1995), suggesting that CalR could have an important role in other cortical cell types during development.

## CONCLUSION

Although many molecular and cellular mechanisms in brain development are shared between humans and rodents, considerable differences stress the need to expand studies of human cortical development. Evolutionary adaptations resulted in the development of the outer SVZ in primates (Smart et al., 2002; Zecevic et al., 2005; Hansen et al., 2010; Lui et al., 2011), which without doubt has a critical role in the expansion and unique organization of the complex human cerebral cortex. The complexity of the CalR^+^ progenitors pool shown here can be translated into a higher diversity of cortical CalR^+^ cells, which might be essential for balanced cortical function. Further studies on the origin and specificity of different interneuron subtypes in the human cerebral cortex are needed to better understand and eventually prevent or treat numerous human-specific psychiatric and neurological disorders.

## Conflict of Interest Statement

The authors declare that the research was conducted in the absence of any commercial or financial relationships that could be construed as a potential conflict of interest.
